# The EPIYA-ABCC motif of Helicobacter pylori cagA gene and gastric carcinogenesis in Casablanca population

**DOI:** 10.4314/ahs.v22i1.66

**Published:** 2022-03

**Authors:** Mohamed R Jouimyi, Ghizlane Bounder, Hasna Boura, Imane Essaidi, Asmaa Bendahmane, Hakima Benomar, Khalid Zerouali, Halima Lebrazi, Anass Kettani, Valérie C Gbonon, Maachi Fatima

**Affiliations:** 1 Laboratory of Helicobacter pylori and Gastric Pathologies, Institut Pasteur du Maroc, Casablanca, Morocco; 2 Gastroenterology Department, National Social Security Fund, Casablanca, Morocco; 3 Laboratory of Histo-Cytopathology, Institut Pasteur du Maroc, Casablanca, Morocco; 4 Microbiology department, Faculty of Medicine and Pharmacy, University Hassan II, Casablanca, Morocco; 5 Department of Bacteriology-Virology, National Reference Center for Antibiotics, Pasteur Institute of Côte d'Ivoire, Abidjan; 6 Laboratory of Biology and Health. Faculty of Sciences Ben M'sik, University Hassan II, Casablanca, Morocco

**Keywords:** cagA gene, EPIYA motifs, gastric carcinogenesis

## Abstract

**Background:**

*H. pylori* infection induce atrophic gastritis (AG) and intestinal metaplasia (IM) that can lead to gastric cancer (GC). The severity of gastric lesions is related to *H. pylori* genetic diversity. The oncogenic potential of H. pylori cagA virulence factor is linked to its high polymorphic EPIYA motifs.

**Objectives:**

Our aim was to evaluate the association of EPIYA motifs with the risk of AG and IM in Casablanca population.

**Methods:**

A total of 210 patients suffering from gastric lesions (chronic gastritis, AG, and IM) was enrolled. H. pylori infection and the type of lesions were diagnosed by ureC PCR and histological examination, respectively. Detection of the cagA gene, and the type of EPIYA motifs, were carried out by PCR

**Results:**

The prevalence of H. pylori and cagA gene was 95% and 37%, respectively. CagA-positive strains were associated with the risk of IM. The EPIYA motifs detected were: EPIYA-ABC (58%), EPIYA-ABCC (22%), and EPIYA-AB (20%). The EPIYA-ABCC motif was associated with the risk of IM (p-value = 0.007), compared to AG (p-value = 0.28).

**Conclusion:**

The EPIYA-ABCC motif might be a useful marker for the identification of patients at high risk of developing IM that can lead to GC.

## Introduction

Helicobacter pylori (*H. pylori*) is a bacterium that colonizes the stomach of nearly half of the world's population^1^. It is now established that long-term *H.pylori* infection is the leading cause of gastric cancer (GC), which develops through gastric carcinogenesis lesions: chronic gastritis (CG), atrophic gastritis (AG), intestinal metaplasia (IM), dysplasia, and GC^2^. The development of gastric lesions is related to a com plex interaction between *H. pylori* virulen ce factors, hu man genetics, and environmental factors. The cytotoxin-associated gene (cagA) is one of the most studied virulence factors of *H. pylori. H. pylori* strains are classified as cagA-positive or cagA-negative strains according to the presence or absence of this gene. Several studies have shown that *H. pylori* cagA-positive strains are associated with high risks of precancerous lesions (AG and IM) and GC^3^–^6^.

The C-terminal variable region of the CagA protein is characterized by a high polymorphic region: the EPIYA (Glu-Pro-Ile-Tyr-Ala) motifs. EPIYA motifs are responsible for CagA downstream effects, and based on the amino acids that flank these motifs, four EPIYA motifs have been identified: EPIYA-A, EPIYA-B, EPIYA-C, and EPIYA-D^7^. The combination of these motifs has been used to classify *H. pylori* cagA-positive strains, and which can be divided into two types: Western cagA type, which possesses EPIYA-A, EPIYA-B, and one to three EPIYA-C motifs, and East-Asian cagA type, which possesses EPIYA-A, EPIYA-B, and EPIYA-D motifs^8^

Phosphorylated CagA, on the tyrosine residue of its EPIYA motifs, interacts with multiple host proteins and triggers abnormal cellular signals, which enhance the risk of damaged cells acquiring precancerous profile. Carrying multiple EPIYA-C repeats or EPIYA-D motif is associated with high risks of precancerous lesions and GC development^9^.

GC is one of the most aggressive neoplasms and it is associated with a poor prognosis. Because of late diagnosis, most Moroccan patients diagnosed are at advanced stages of the disease, which result in a five-year survival rate less than 15%^10^. Therefore, finding a marker for the risk of developing this cancer is an important step in reducing its mortality. The aim of our study was to identify the type of EPIYA motifs circulating in a population of the Casablanca city, as well as to investigate their association with the risk of developing precancerous lesions, in order to use it as predictive markers in the identification and follow-up of patients that present a high risk to develop precancerous lesions and GC.

## Material and methods

### Study population

A total of 210 patients consulting in the gastroenterology service of the National Social Security Fund of Casablanca, Morocco and suffering from digestive pain were included in this study. From all patients, 6 biopsies (2 from the antrum, 2 from the fundus, and 2 from the lesser curvature) have been sampled. Three biopsies (1 from the antrum, 1 from the fundus, and 1 from the lesser curvature) were used for histological examination and the other three biopsies were used for molecular detection.

All participants were informed of their inclusion in the study and agreed to it on a writing form. The present study has been performed in accordance with the ethical standard of the 1964 Declaration of Helsinki.

### Histology

The biopsy samples were transported in 10% formalin and embedded in paraffin and multiple histological sections were obtained from each biopsy. Biopsy sections were then stained with hematoxylin-eosin for the detection of gastric lesions. The blades were read by a pathologist.

### PCR for *H. pylori* detection

Total DNA was extracted from gastric biopsies using a genomic DNA extraction kit (Isolate Genomic DNA Kit, BioLine). Using primers described by Lu^11^, the ureC gene (296 bp) was amplified to detect H. pylori infection. The PCR reaction mixture was prepared with 0.5 mM dNTPs, 1.5 mM MgCl2, 0.5 µM of each primer, 1 U of DNA Polymerase (MyTaq DNA Polymerase, BioLine) and 300 ng of DNA in a final volume of 20 µL. PCR thermocycling conditions were as follows: 1 cycle at 95°C for 1 min, 35 cycles at 95°C for 15 s, 55°C for 30 s, 72°C for 30 s and a final extension cycle at 72°C for 7 min.

### CagA gene and variable region amplification

H. pylori positive samples were subjected to the detection of the cagA gene by PCR using primers described by Yamaoka^12^. To amplify the 550 to 850 bp region within the 3′ variable region of the cagA gene, the primers cag2F and cag4 described by Argent were used^13^.

The PCR reaction mixture was prepared with 0.5 mM dNTPs, 1.5 mM MgCl2, 0.2 µM of each primer, 1 U of DNA Polymerase (MyTaq DNA Polymerase, BioLine) and 300 ng of DNA in a final volume of 20 µL. The PCR products were separated by electrophoresis on a 1.5% agarose gel, followed by ethidium bromide staining and UV light analysis. PCR thermocycling conditions are shown in table 2.

**Table 2:** Distribution of gastric lesions according to age and gender

	CG n = 129 (%)	AG n = 53 (%)	IM n = 28 (%)	p-value
Age (mean ± sd)	48 ± 17	49 ± 13	53 ± 17	0.39 *
Gender Males Females	62 (48) 67 (52)	19 (36) 34 (64)	18 (64) 10 (36)	0.04 **

CG: chronic gastritis, AG: atrophic gastritis, IM: intestinal metaplasia, sd: standard deviation.

* ANOVA test

** Chi-square test.

### EPIYA motifs amplification

Each cagA-positive sample was subjected to 4 PCR reactions to identify the type of EPIYA motifs. The cag28F was used as former primer in all 4 reactions, while the reverse primers cagAP1C, cagAP2TA, cagA west, cagA east, were used in separate reactions to amplify the EPIYA-A, EPIYA-B, EPIYA-C, and EPIYA-D motifs, respectively^13^,^14^.

For the EPIYA-A, EPIYA-B, and EPIYA-C motifs, the reaction mixture was as described for the cagA gene. For the amplification of the EPIYA-D motif, the PCR reaction mixture was prepared with 1 mM dNTPs, 3 mM MgCl2, 0.2 µM of each primer, 1 U of MyTaq DNA Polymerase (MyTaq DNA Polymerase, BioLine) and 300 ng of DNA in a final volume of 20 µL. PCR thermocycling conditions are shown in Table 1.

**Table 1:** PCR thermocycling conditions used in this study

Motif amplified	PCR thermocycling conditions
*cagA* gene constant region	1 cycle at 95°C for 1 min, 35 cycles at 95°C for 15 s, 58°C for 30 s, 72°C for 30 s and a final extension cycle at 72°C for 7 min
*cagA* variable region	1 cycle at 95°C for 1 min, 35 cycles at 95°C for 15 s, 55°C for 30 s, 72°C for 30 s and a final extension cycle at 72°C for 7 min
EPIYA-A motif	1 cycle at 95°C for 1 min, 35 cycles at 95°C for 15 s, 57°C for 30 s, 72°C for 30 s and a final extension cycle at 72°C for 7 min
EPIYA-B motif	1 cycle at 95°C for 1 min, 35 cycles at 95°C for 15 s, 56°C for 30 s, 72°C for 30 s and a final extension cycle at 72°C for 7 min
EPIYA-C motif	1 cycle at 95°C for 1 min, 35 cycles at 95°C for 15 s, 61°C for 30 s, 72°C for 30 s and a final extension cycle at 72°C for 7 min
EPIYA-D motif	1 cycle at 95°C for 1 min, 35 cycles at 95°C for 15 s, 59°C for 30 s, 72°C for 30 s and a final extension cycle at 72°C for 7 min

### Statistical analysis

The statistical analysis were conducted using R software version 3.4.0. A univariate analysis using Chi-square, Fisher, and ANOVA tests was performed to assess all associations between gastric lesions, age, gender, cagA-gene status, and EPIYA motifs.

For the association between gastric lesions and cagA-gene status, gastric lesions were considered as the dependent variable and cagA-gene status as the predictor variable. The CG group and cagA-negative strains were taken as reference.

For the association between gastric lesions and EPIYA motifs, gastric lesions were considered as the dependent variable and repetition of EPIYA-C motif as the predictor variable. The CG group and EPIYA-AB motif were taken as reference.

Results were expressed as odds ratio (OR), 95% confidence intervals (95% CI) and p-values.

## Results

### Population characteristics

The population is constituted by 99 (47%) males and 111 (53%) females. The mean age of the population was 49 ± 16. Of the 210 studied patients, 61% were diagnosed with CG, 25% with AG, and 13% with IM. Gastric lesions severity was increasing with age, but without being statistically meaningful (p-value = 0.39). Concerning gender, frequency of females and males diagnosed with CG was the same. Females were more diagnosed with AG than males, but this observation is reversed in the case of IM (Table 2). Association between gender and gastric lesions severity was statistically significant (p-value = 0.04).

The presence of H. pylori was detected in 200 patients (95%) as follow: 121 cases (94%) in CG, 51 cases (96%) in AG, and 28 cases (100%) in IM.

### Distribution of the cagA gene according to gastric lesions

Of the 200 H. pylori-positive patients, the cagA gene was detected in 74 cases (37%). The frequency of the cagA-positive strains was found to increase according to gastric lesions severity: 34/121 in CG (28%), 22/51 in AG (43%), and 18/28 in IM (64%). Distribution of the cagA gene among gastric lesions was statistically significant (p-value < 0.001).

### Association between the cagA gene and the risk of gastric precancerous lesions

According to table 3, the frequency of cagA-positive strains was higher in AG (43%), compared to CG (28%). In contrast, the frequency of cagA-negative strains was lower in AG (57%), compared to CG (72%). However, infection with cagA-positive strains was not associated with the risk of AG (OR = 1.94, 95% CI = 0.98 – 3.83, p-value = 0.07).

**Table 3:** Association between cagA gene and precancerous lesions development

	*CagA*-positive n (%)	*CagA*-negative n (%)	OR	95% CI	p- value
CG	34 (28)	87 (72)	-	-	-
AG	22 (43)	29 (57)	1.94	0.98 – 3.83	0.07
IM	18 (64)	10 (36)	4.6	1.9 – 10.97	< 0.001

CG: chronic gastritis, AG: atrophic gastritis, IM: intestinal metaplasia. OR: odds ratio, 95% CI: 95% confidence interval.

In IM, a very high frequency of cagA-positive strains was found between IM and CG (64 and 28%, respectively), while a high frequency of cagA-negative strains was found in CG compared IM (72 and 36%, respectively). Therefore, infection with cagA-positive strains was associated with IM, and increases its risk by an OR of 4.6 (95% CI = 1.9 – 10.97, p-value < 0.001).

### Detection of cagA variable region and typing of EPIYA motifs

Of the 74 cagA-positive strains, the cagA variable region was detected in 69 cases (93%). The 3 variable region of the cagA-positive strains showed different EPIYA motifs: 40 EPIYA-ABC (58%), 15 EPIYA-ABCC (22%), 14 EPIYA-AB (20%). No case of EPIYA-D was detected.

### Distribution of EPIYA motifs according to gastric lesions

The frequency of the EPIYA-AB motif was slightly elevated in CG (24.1%) compared to AG and IM (18 and 17%, respectively). The frequency of the EPIYA-ABC motif decreased according to gastric lesions severity: 72.4% in CG, 64% in AG, and 28% in IM. The frequency of the EPIYA-ABCC motif increased according to gastric lesions severity: 3.4% in CG, 18% in AG, and 55% in IM (Table 4).

**Table 4:** Distribution of EPIYA motifs according to gastric lesions

	CG n = 29 (%)	AG n = 22 (%)	IM n = 18 (%)
EPIYA-AB motif	7 (24.1)	4 (18)	3 (17)
EPIYA-ABC motif	21 (72.4)	14 (64)	5 (28)
EPIYA-ABCC motif	1 (3.4)	4 (18)	10 (55)

CG: chronic gastritis, AG: atrophic gastritis, IM: intestinal metaplasia.

### Association between EPIYA-C repetition and the risk of gastric precancerous lesions

According to table 5, the frequency of EPIYA-AB and EPIYA-ABC motifs was the same in CG and AG. Thus, no association was found between the EPIYA-ABC motif and the risk of AG (OR = 1.16, 95% CI = 0.28 – 4.74, p-value = 1). In IM, the frequency of EPIYA-AB motif was elevated (37.5%) compared to CG (25%), while the frequency of the EPIYA-ABC motif was lower (62.5%) compared to CG (75%). Therefore, the EPIYA-ABC motif was not associated with the risk of IM (OR = 0.58, 95% CI = 0.1 – 3.09, p-value = 0.66).

**Table 5:** Association between EPIYA-ABC motif and precancerous lesions development

	EPIYA-AB motif n (%)	EPIYA-ABC motif n (%)	OR	95% CI	p- value
CG	7 (25)	21 (75)	-	-	-
AG	4 (22)	14 (78)	1.16	0.28 – 4.74	1
IM	3 (37.5)	5 (62.5)	0.58	0.1 – 3.09	0.66

CG: chronic gastritis, AG: atrophic gastritis, IM: intestinal metaplasia, OR: odds ratio, 95% CI: 95% confidence interval.

According to table 6, the frequency of the EPIYA-AB motif was higher in CG (87.5%) compared to AG (50%), while the frequency of the EPIYA-ABCC motif was lower in CG (12.5%) compared to AG (50%). However, no association was found between the EPIYA-ABCC motif and the risk of AG (OR = 7, 95% CI = 0.56 – 86.32, p-value = 0.28). In IM, the frequency of the EPIYA-AB motif was very low (23%) compared to CG (87.5%), while the frequency of the EPIYA-ABCC motif was very high (77%) compared to CG (12.5%). Therefore, the EPIYA-ABCC motif was associated with the risk of IM (OR = 23, 95% CI = 1.99 – 273.29, p-value = 0.007).

**Table 6:** Association between EPIYA-ABCC motif and precancerous lesions development

	EPIYA-AB motif n (%)	EPIYA-ABCC motif n (%)	OR	95% CI	p-value
CG	7 (87.5)	1 (12.5)	-	-	-
AG	4 (50)	4 (50)	7	0.56 – 86.32	0.28
IM	3 (23)	10 (77)	23	1.99 – 273.29	0.007

CG: chronic gastritis, AG: atrophic gastritis, IM: intestinal metaplasia, OR: odds ratio, 95% CI: 95% confidence interval.

## Discussion

In our population, the prevalence of cagA-positive strains was 37%. Our finding is in accordance with a Moroccan and Kenyan study^15^,^16^, but differs from other African studies^17^–^22^. In contrast, our rate is different from that found by another Moroccan study conducted in the city of Fes, where the prevalence of the cagA gene was 69%^23^. This difference can be explained by the high incidence of GC encountered in this city compared to our city (Casablanca), as reported by the study done by Fadloullah et al 24. In addition, the same observations have been reported in several countries, such as Mexico (43 and 90%) and Turkey (52 and 94%)^25^,^26^.

An increasing rate of cagA-positive strains with gastric lesions severity was noted. However, the association between cagA-positive strains and precancerous lesions was most significant in the case of IM than AG (Table 3). This finding has been previously shown by several studies, which demonstrate the carcinogenic potential of H. pylori cagA-positive strains^27^–^30^.

According to our results, the Moroccan *H. pylori* cagA-positive strains belong to the Western motif which is characterized by the presence of the EPIYA-C motif, similar to African^20^,^23^, European^31^,^32^, and American populations^25^,^30^,^33^. Furthermore, and along with the above studies, we did not find any case of East-Asian motif (EPIYA-D motif), which appears to be an endemic motif of the Asian continent, except in some countries, where both EPIYA-C and EPIYA-D motifs exist^34^,^35^.

The EPIYA-ABC motif was the most encountered motif (57%) in our area. Based on other African studies^20^,^23^, it seems that the EPIYA-ABC motif is the most circulating motif in our continent. However, other reports are needed to confirm this hypothesis since African studies on cagA EPIYA motifs are rare. The EPIYA-ABCC motif was the second most encountered motif (20%). Similar rates have been reported in different populations: Brazil (23.1%), Iran (25.6%), Bangladesh (26.3%)^36^–^38^. In contrast, a Moroccan study reported a low frequency of the EPIYA-ABCC motif (9.8%)^23^. In their study, El-khadir et al included patients suffering from different gastric diseases (CG, peptic and duodenal ulcers, and GC), while our study included patients suffering from gastric carcinogenesis lesions (CG, AG, and IM). Difference in study design may explain the different results observed.

In the present study, we investigated the relationship between gastric mucosal lesions severity spanning multiple stages of gastric carcinogenesis with the number of EPIYA-C motifs. Our results showed that the risk of developing IM increases with the increasing number of EPIYA-C motif (Tables 5 and 6). Our findings are in accordance with those from studies on Italian, Colombian, and Brazilian populations, in which cagA strains harboring high numbers of EPIYA-C motifs were associated with IM^31^,^33^,^36^. This can be explained by the fact that CagA proteins with more repetitions of EPIYA-C motif are known to have a greater affinity with SHP2 protein compared to CagA proteins with one EPIYA-C motif, which rapidly increase the evolution of gastric lesions towards more severe lesions such as IM^39^.

On the other hand, no association was found between the increasing number of EPIYA-C motif and AG, even though the risk of developing this lesion remains high in the case of EPIYA-ABCC motif compared to EPIYA-ABC motif (Tables 5 and 6). Reports addressing repetitions of EPIYA-C motif and development of AG have shown divergent results. For example, studies done on Colombian populations did not find an association between the increasing number of EPIYA-C motif and AG^33^,^40^. In contrast, reports fromrazilian and Portuguese populations could demonstrate this association^32^,^36^. These differences could be explained by the study design. In the Brazilian study, all included patients were infected with cagA-positive strains, while in the Portuguese study, from the 21 patients that had AG, 20 patients had also IM and only one patient had only AG^32^,^36^.

In conclusion, the results of our study showed that the cagA EPIYA motifs from Casablanca city belong to Western strains, and the EPIYA-ABC motif being the most encountered motif. In addition, infection with H. pylori cagA EPIYA-ABCC strains represent a risk factor for IM development. Therefore, identification of the type of cagA EPIYA motifs may be used as a prediction tool for identification of patients at a high risk of developing IM which is considered as a precursor of GC development.
